# Supplementary material for: Are carbon emissions trading and green financial instruments synergistic? -Comprehensive quantitative research based on content analysis
Source: PLoS One. 2024 Mar 7;19(3):e0298601. doi: 10.1371/journal.pone.0298601 (PMC10919601; doi:10.1371/journal.pone.0298601)
Supplement: S1 Data — (ZIP) [file pone.0298601.s001.zip › Data/地级市Z_F工作报告.zip!/╣π╢1⁄2/╣π╓▌╩╨/└┤╫╘╣1⁄2╓┌║┼í╛┬φ┐╦╩2╛▌═°í┐_2018.docx]

各位代表：

现在，我代表市人民政府，向大会报告政府工作，请予审议，并请政协各位委员和其他列席人员提出意见。

一、2017年工作回顾

过去一年，我们在以习近平同志为核心的党中央坚强领导下，全面贯彻落实省委、省政府和市委决策部署，坚持稳中求进工作总基调，坚持新发展理念，统筹推进稳增长、促改革、调结构、惠民生、防风险各项工作，较好完成了市十五届人大一次会议确定的主要目标任务。

一年来，我们坚持把迎接党的十九大、学习宣传贯彻党的十九大精神，作为全年工作的主线，坚持用习近平总书记对广东工作重要指示批示精神统领各项工作，在学懂弄通做实党的十九大精神上狠下功夫，推动习近平新时代中国特色社会主义思想在广州落地生根、结出丰硕成果。

去年，我市经济社会发展稳中向好。发展动能转换成效明显，全市地区生产总值突破2万亿元，高新技术企业快速发展，一批世界500强企业重大项目落户广州。城市枢纽功能更加强化，机场、港口、轨道交通等建设取得突破性进展，市容市貌大幅提升。营商环境更加优越，放管服改革持续深化，新一轮城市总体规划编制等一批国家级试点全面铺开，广州的影响力、显示度不断增强。人民生活持续改善，十件民生实事全面兑现，房地产市场保持平稳，空气质量位居全国大城市前列，＂广州蓝＂成为一张靓丽的城市名片。去年，我们成功举办《财富》全球论坛，为构建全球经济新格局贡献了广州智慧和广州力量。

一年来，主要做了以下工作：

（一）努力提高经济发展质量和效益。面对经济下行压力，我们保持战略定力和战略耐心，致力于打基础、解难题、谋长远，把工作重点放在转变发展方式、优化经济结构、转换增长动力上，经济保持平稳发展。

经济实力稳步提升。大力实施＂攻城拔寨＂行动，全力推进345个重点项目建设，加快发展实体经济，大力拓展海外市场，推动发展动能转换，有力促进经济增长。预计全年地区生产总值2.15万亿元，比上年增长7％以上。完成固定资产投资6000亿元，增长8％左右。社会消费品零售总额9400亿元，增长8％。外贸进出口总值9600亿元，增长12％。实际使用外资62.89亿美元，增长10.3％。来源于广州地区的一般公共预算收入5947亿元，增长14％，其中地方一般公共预算收入1533亿元，可比增长10.9％。城乡居民人均可支配收入分别增长8.5％和9.5％左右。城市居民消费价格指数上涨2.3％。年末地区本外币存款余额5.14万亿元，增长8.1％；贷款余额3.41万亿元，增长15.1％。

创新步伐明显加快。加大科技创新扶持力度，全社会研发投入增幅超过20％，占地区生产总值2.5％左右。坚持数量扩张与质量提升并举，下大力培育发展科技创新主体，新增高新技术企业4000多家、科技企业孵化器面积69万平方米、众创空间49家，获批成立再生医学与健康省实验室、国家先进高分子材料产业创新中心，美国冷泉港实验室、斯坦福国际研究院落户。举办中国创新大会、创交会、海交会、小蛮腰科技大会、风险投资论坛等高端会议，科技信贷风险补偿资金池撬动银行贷款60亿元。加强创新人才队伍建设，累计发放人才绿卡3300张，新入选国家＂千人计划＂专家65人，引进创新创业领军团队21个、各类领军人才58人。专利申请量增长35％左右，专利行政执法绩效考核全国第一，广州成为首批国家知识产权强市创建市。

新兴产业不断壮大。出台发展先进制造业、现代服务业、战略性新兴产业等19份政策文件，设立产业引导基金，举办人工智能、生物产业等大会，高起点布局发展IAB和NEM产业。坚持产业集聚集约集群发展，重点培育5个千亿级工业产业集群，建设10个价值创新园区，琶洲互联网创新集聚区初具规模，富士康超视堺、乐金8.5代OLED、思科智慧城、广汽智能网联、通用生物产业园、百济神州、粤芯芯片等重大项目加快建设。实施新一轮工业技术改造，工业企业利润增长13％。全市高新技术产品产值增长9％，战略性新兴产业增长10％，先进制造业增加值占制造业比重64％左右，现代服务业增加值占服务业比重66％，产业向高端高质高新发展迈出一大步。

＂三去一降一补＂取得成效。积极化解落后产能，全面取缔＂地条钢＂，完成466户国有＂僵尸企业＂出清重组。强化债务风险管理，开展互联网金融整治，银行机构不良贷款率1.1％，低于全国、全省平均水平，债务率低于警戒线，风险总体可控。切实降低企业成本，落实税收优惠政策，取消、停征和降低47项行政事业性收费，全年为企业减负超过800亿元。实施质量强市战略，制造业产品质量合格率96.74％，省名牌申报数量增长80.9％。推进农业供给侧结构性改革，加快89宗农村水利工程加固和20项防洪工程建设，国际种业中心建设取得新进展，本市产地农产品质量稳定优于国家标准。

（二）在更高起点上推动改革开放。贯彻落实习近平总书记关于北上广深等特大城市要率先加大营商环境改革力度的重要指示精神，把改革开放放在更加突出的位置，努力打造全球企业投资首选地和最佳发展地。

成功举办《财富》全球论坛。习近平总书记致贺信，引起全球热烈反响。2万多家媒体参与宣传报道，组织13场全球路演活动，与会企业388家，中外嘉宾1100多人，论坛规模超过历届，《财富》国际科技头脑风暴大会永久落户广州。论坛充分展示了广州开放创新的新形象，提升了市民群众的自豪感和荣誉感，扩大了广州在国际上的知名度和影响力。

重点领域关键环节改革取得新进展。推进＂中国制造2025＂等50项国家和省的改革试点，绿色金融改革总体方案获国务院批准实施，中新广州知识城获批成为中新国家级双边合作项目，市场采购规模跃居全国第二。深化国资国企改革，国有资本运营公司试点初见成效，完成4户一级企业整合重组，推进4户企业员工持股省级试点，国有企业利润总额增长10％以上，国有资产保值增值率113％以上。出台促进民营经济发展20条，竞争性领域价格基本放开，私营企业主体增长26.3％。深化国税、地税征管体制改革，62项重点任务全面完成。教育、医疗、卫生、养老、社保等公共服务领域改革持续深化。农村综合改革有力推进，超额完成农村土地承包经营权确权登记颁证年度任务。

营商环境持续优化。承接省级行政职权事项120项，实施放权强区改革，下放143项行政职权事项和126项公共服务事项，完善市、区财政体制，激发和调动各区积极性。实施投资贸易便利化措施124项，精简行政许可备案事项92项，清理规范审批中介服务217项，26类建设项目免于环评审批。深化商事制度改革，推行全程电子化商事登记模式，新登记市场主体增长33.9％，日均增加898户。完善国际贸易单一窗口，推进＂三互＂大通关建设，推出＂互联网＋易通关＂、智检口岸、全球质量溯源体系等改革，货物通关时间压减一半。南沙自贸区新增制度创新成果101项，有23项在全国、全省复制推广。广州在世界级城市体系、国际金融中心和城市竞争力等排名稳步提升。

开放合作力度加大。以＂一带一路＂建设为引领，全面深化中新、中欧、中以、中沙、中瑞等国际合作，打造一批中外合作产业园区，加快构建全球投资贸易网络，与沿线国家和地区贸易额增长24.64％。在美国硅谷、波士顿和以色列特拉维夫成立科技办事处，新增6个国际友好城市、3个国际友好港。成功举办从都国际论坛、国际投资年会、夏季达沃斯＂广州之夜＂、世界城市日全球主场活动、国际金融论坛全球年会等47场国际性会议，成功申办2019年世界港口大会、2020年世界大都市协会世界大会。启动粤港深度合作区起步区建设，实施粤港澳游艇＂自由行＂。深入推动广佛肇清云韶经济圈建设，开展与东莞、中山等市的战略合作。做好精准扶贫工作，扎实推进援疆援藏和梅州、清远、毕节、黔南、巫山、甘孜对口帮扶，推进＂百企扶百村＂行动，市财政落实扶贫资金21.62亿元。

（三）高水平推进城市规划建设管理。尊重城市发展规律，对标国际一流城市，把广州的发展放到全球资源配置体系中谋划，不断强化城市枢纽网络功能。

规划引领作用更加凸显。围绕＂两个一百年＂奋斗目标，高水平编制面向2035年的城市总体规划，研究论证新时代城市发展理念、目标定位、空间格局、功能布局，描绘城市未来发展的宏伟蓝图。城市规划展览中心实现开馆试运营。推进广州南站、珠江国际慢岛、广船地块等城市设计深化工作，获批全国城市设计试点。调整完善土地利用总体规划，新增105平方公里建设用地规模，突出重点项目和民生用地保障，加大批而未供、供而未用土地整治力度，国土资源集约节约利用水平显著提高。

交通基础设施建设进度加快。围绕打造世界级枢纽港区，大力推进港口基础设施建设，南沙港三期建成投产，广州港深水航道拓宽一期工程主体完工，新增12条国际班轮航线，港口货物吞吐量5.9亿吨，集装箱吞吐量2035万标箱，稳居世界前列。加快白云机场扩建，第二航站楼全面完工，商务航空服务基地投入使用，开展第二机场选址，建设临空经济示范区，白云机场旅客吞吐量6580万人次，排名全国第三。加快综合交通枢纽建设，统筹推进一批国铁、城际轨道、高速公路、国省道主干线和市政路桥工程建设，广州大桥拓宽工程顺利完工，新开通地铁4条（段）、总运营里程近400公里，大交通网络体系加快形成。

生态环境明显优化。铁腕整改中央环保督察发现的问题，841件市民群众反应强烈的问题得到解决。坚决打赢蓝天保卫战，扩大高污染燃料禁燃区，全市限行黄标车，新增纯电动公交车2433辆，完成545座加油站、13座储油库油气回收系统整改，PM2.5年平均浓度35微克/立方米，在人口规模超千万、经济总量超万亿的国家中心城市中率先达到国家标准。全力打响水污染防治攻坚战，全面推行河长制，强力推进＂四洗＂治水行动，清除污染源3.7万个，整治2033个排水口，开工建设48条城中村截污纳管，完成316条行政村生活污水治理，新建成污水处理厂5座、污水管网1392公里，新增污水处理能力19万吨/日，全市整体水质明显改善，国家督办的35条黑臭河涌治理达到初见成效考核要求。推行生活垃圾强制分类，开展示范街镇和居住小区（社区）创建活动，优化分类收运体系，关停并转＂洋垃圾＂加工企业23家，5座资源热力电厂点火烘炉。开展农用地土壤污染详查。实施森林品质和园林景观提升工程，基本完成国有林场改革，举办国际花卉艺术展，种植开花乔木10万株，新增森林公园6个、湿地公园3个、生态景观林带80公里，建成区绿化覆盖率42.54％，人均公园绿地面积17.06平方米，城市绿化水平稳步提升。

城市更新取得进展。制定城市更新三年用地保障计划，保障供地9.26平方公里。设立城市更新基金，实施差异化改造政策。推进109个老旧小区微改造、13条旧村全面改造、4个村级工业园转型升级，广氮、广船地块等重点项目落地实施，老旧小区微改造列入国家试点，经验在全国推广。开展新型城镇化综合试点，推进北部山区30个特色小镇、74条美丽乡村、1个名镇、15条名村创建，番禺沙湾镇入选全国特色小镇。

城市管理更加精细。推进城市环境品质提升行动，完成6大类300多个道路环境绿化景观项目，清洗高快速路附属设施4122公里，城市路更新、花更美、灯更靓。重拳出击违法建设，专项整治户外广告招牌23万平方米，查处违法建设1088万平方米，整治出租屋安全隐患60.2万处。推进6个地下综合管廊建设，完成8.68万公里地下管线安全隐患排查，新增管道燃气用户16万户，超算中心天然气分布式能源站建成投运。健全应急管理体系，科学防范应对突发事件。健全安全生产治理体系，完善安全生产责任制，开展重点行业领域专项治理，生产安全事故起数、死亡人数分别下降9.3％和11.8％。

（四）不断提升市民群众的获得感幸福感安全感。始终把人民利益摆在至高无上的地位，在财政支出中持续加大民生投入，一般公共预算用于民生支出占比达到76.8％。

社会事业全面发展。大力发展教育事业，推动中小学校三年提升计划，新增普惠性幼儿园82所，新改扩建中小学43所、新增学位5.2万个，新建示范性普通高中10所，成立19个教育集团，引进基础教育名教师名校长53名、高等教育高层次人才616名，成功创建省推进教育现代化先进市。推动高水平大学建设，广州地区5所高校入选＂双一流＂，部省市校四方签约建设华南理工大学广州国际校区。推动职业教育发展，世界技能大赛我市选手获2金1银3铜，奖牌数位居全国前列。积极促进就业，举办就业招聘会、就业技能培训和就业援助活动，新增就业33万人，城镇登记失业率2.4％。建设卫生强市，启动152家公立医院改革，全面取消药品加成，新组建39个医联体，对儿科医疗实行专项经费补助，有效应对禽流感、登革热等传染病疫情。加强农产品和食品药品监管。大力发展体育事业，成功举办金砖国家运动会、广州马拉松赛等重大体育赛事，全运会获18枚金牌，体育场馆惠民1000多万人次。扩大来穗人员基本公共服务覆盖面，完成6000人积分制入户，安排随迁子女学位2.5万个。

文化建设扎实推进。深入推进社会主义核心价值观建设，群众性精神文明创建活动蓬勃开展，蝉联全国文明城市。整合举办广州文化产业交易会，建成78个文化广场，开展群众性文化活动1.8万场，南粤先贤馆开馆运行，粤剧艺术博物馆荣获国家优质工程鲁班奖。成立全国首个文化上市公司产业联盟，设立100亿元文化产业投资基金，城市文化竞争力指数稳居全国第三。促进商旅文融合，召开旅游发展大会，推出旅游扶持政策，完善旅游公共服务，荣获全国旅游厕所革命先进市。

社会保障体系进一步完善。坚持＂房子是用来住的、不是用来炒的＂定位，综合运用土地、信贷等措施加强房地产调控，供应住宅用地4.57平方公里，房地产市场没有大起大落。率先实施＂租购并举＂，组建3家国有住房租赁公司，搭建＂阳光租房＂平台，住房租赁市场加快发展。加大保障房建设力度，推出各类保障房3.1万套，中等偏下收入住房困难家庭应保尽保，2.6万名新就业无房职工首次纳入保障。推行城乡居民医疗保险全覆盖，实现跨省医保异地就医网上实时结算，开展长期护理保险制度试点，城乡居民大病保险最高支付限额提高至45万元，企业职工月人均养老保险金提高至3471元。加大困难群体帮扶力度，开展医疗救助83.55万人次，城乡低保标准每人每月提高到900元，保障水平位居全国前列。加强双拥优抚安置工作，提高优抚安置对象保障水平。支持社会力量举办养老机构，每千名老人拥有养老床位数40张。政府出资为所有户籍老人购买意外伤害综合保险。深化社区居家养老服务改革，设立养老服务助餐配餐点846个，镇街、村居覆盖率100％，让老年人生活更舒心，让年轻人工作更安心。

社会治理得到加强。圆满完成村居换届选举工作。完善基层网格化管理，提升精细化服务管理能力。全面推行城乡社区协商自治，开展协商议事1.2万次、办结率94.25％。创新推动＂社工＋＂战略，社工服务群众300万人次。开展社会组织公益创投，资助公益项目165个。成立全国首个＂慈善之城＂创建联盟，社会认捐款物达5.45亿元。开展＂四标四实＂工作，搭建基础数据平台。深入推进＂雪亮工程＂建设，城市主要道路和重点区域实现视频监控全覆盖。圆满完成党的十九大、《财富》全球论坛等系列重大安保维稳工作。创新打击违法犯罪工作机制，全市案件类警情、刑事立案同比分别下降12.2％和17.7％，市民安全感和满意度进一步提升。推进公共法律服务最便捷城市建设，普法、社区矫正、人民调解、监狱和戒毒管理工作进一步加强。少数民族服务管理体系不断完善，宗教团体和场所规范化管理水平明显提升。

此外，军民融合发展、国防动员、人民防空、海防打私、打假、妇女儿童、残疾人、侨务、对台、贸促、气象、地震、统计、参事、文史、档案、保密、供销、修志、区划地名、仲裁、红十字会等工作取得新进步。

（五）努力建设为民务实清廉政府。一年来，我们始终贯彻全面从严治党要求，把政治纪律和政治规矩挺在前面，牢记宗旨，勤勉尽责，依法行政，公正用权，增强政府执行力和公信力，努力为人民提供优质高效服务。坚持向人大及其常委会报告工作，向人民政协通报情况，主动依法接受监督，习惯在监督下开展工作，善于在监督中积极有为。办理市人大代表议案决议2件、市政协建议案6件，办理各级人大代表建议322件、政协提案343件。认真听取民主党派、工商联、无党派人士及工会、共青团、妇联等人民团体意见。加强法治政府建设，提请市人大常委会审议法规草案7件、制定修改政府规章8项，清理法规、规章、规范性文件1030件，废止规章、规范性文件194件。开展行政执法＂三项制度＂试点，创新行政复议机制，办理行政复议案件2007件。荣获全国法治政府建设典范城市称号。实施市政府部门定期新闻发布会制度，开展基层政务公开标准化规范化试点，完善政务公开考核机制，率先向社会公示行政执法数据，财政透明度位居全国前列。推行＂互联网＋政务服务＂，实现审批服务事项一窗受理、网上办理和部分事项全城通办，不动产登记实现登记与税务一体办理，通过12345热线推动解决市民诉求114万多宗，市民满意度不断提升。依法及时就地解决群众合法诉求，全年办理群众信访事项1.9万件次，信访积案化解率达100％。严格落实＂一岗双责＂，驰而不息正风肃纪，坚定不移惩治腐败，强化政府投资项目廉情预警评估。深化领导干部经济责任审计，廉洁风险防控进一步加强，廉洁政府建设取得新成效。

各位代表，过去一年的成绩来之不易，这是以习近平新时代中国特色社会主义思想为指导的结果，是省委、省政府和市委坚强领导的结果，是全市人民凝心聚力、团结奋斗的结果。我代表广州市人民政府，向全市人民、驻穗部队官兵、各民主党派、人民团体以及社会各界人士表示崇高的敬意！向关心支持广州改革发展的港澳台同胞、海外侨胞、国际友人表示衷心的感谢！

我们也清醒认识到，政府工作还存在不少挑战和薄弱环节。去年政府工作报告分解的218项重点工作，还有个别指标和任务完成不理想。经济社会发展不平衡不充分的问题依然存在：一是经济发展质量有待提高，工业增长乏力，创新型龙头企业不强，企业研发投入不高，传统产业转型升级不够快，新兴产业还未形成有力支撑，民间投资不足，民营经济发展不充分。二是城市规划科学性前瞻性还需提升，城市建设有待加强，工程项目统筹协调力度不够，一些重点项目推进较慢，立体交通网络体系有待完善，城市更新任务艰巨，农村基础设施建设还有不少欠账。三是城市管理不够精细科学，部分城乡结合部、城中村＂脏乱差＂问题仍然突出，安全生产和消防存有隐患，污染治理压力较大，道路交通安全和拥堵问题仍较突出，应对电信、网络等新领域突出问题的能力有待提升。四是基本公共服务均等化水平仍需提高，就业、教育、医疗、住房、养老等方面与群众期待还有差距。五是政府职能转变不够到位，改革的力度和深度不够，群众办事还有诸多不便，少数干部服务意识不强，不想为、不敢为的现象仍然存在，一些领域腐败案件时有发生。这些问题，我们必须着力加以解决。

二、2018年工作安排

今年是贯彻党的十九大精神的开局之年，是改革开放40周年，是决胜全面建成小康社会、实施＂十三五＂规划承上启下的关键一年。做好今年的政府工作，我们必须坚决维护以习近平同志为核心的党中央权威和集中统一领导，不折不扣贯彻落实以习近平同志为核心的党中央决策部署，在省委、省政府和市委的正确领导下，突出抓重点、补短板、强弱项，努力开创政府工作新局面。

今年政府工作总体要求是：全面贯彻党的十九大和中央经济工作会议、中央农村工作会议精神，以习近平新时代中国特色社会主义思想为指导，按照＂三个定位、两个率先＂和＂四个坚持、三个支撑、两个走在前列＂的要求，坚持稳中求进工作总基调，坚持新发展理念，坚持高质量发展要求，紧扣我国社会主要矛盾变化，统筹推进＂五位一体＂总体布局，协调推进＂四个全面＂战略布局，以供给侧结构性改革为主线，持续推进枢纽型网络城市建设，更加注重经济发展质量，更加注重城乡发展质量，更加注重人民美好生活质量，充分发挥国家重要中心城市引领和辐射带动作用，努力在决胜全面建成小康社会、加快建设社会主义现代化新征程上走在前列。

今年经济社会发展主要预期目标是：地区生产总值增长7.5％左右，固定资产投资增长11％左右，社会消费品零售总额增长7.5％左右，商品出口总值增长5％左右。经济结构和质量效益不断提升，先进制造业增加值占制造业比重达到66％左右，现代服务业增加值占服务业比重达到67％左右，研发经费支出占地区生产总值比重达到2.7％，地方一般公共预算收入可比增长7.5％。人民生活水平进一步提高，城乡居民收入增长与经济增长基本同步，城市居民消费价格涨幅控制在3％左右，城镇登记失业率控制在3.5％以内。生态环境持续改善，万元地区生产总值能耗下降4％，万元地区生产总值用水量下降4％，节能减排降碳约束性指标完成年度任务。金融、财政风险得到有序有效防控。为实现上述目标，我们着力在以下几个方面狠抓落实：

（一）深化供给侧结构性改革。围绕建设现代化经济体系，把发展经济的着力点放在实体经济上，把提高供给体系质量作为主攻方向，坚定不移加快产业转型升级，积极配置全球高端资源要素，促进新动能快速成长，推动经济发展质量变革、效率变革、动力变革。

推动先进制造业集聚发展。加快产业结构调整，力争工业投资1500亿元，增长100％。开展＂中国制造2025＂试点示范城市建设，制定实施IAB产业发展五年行动计划。推动协同创新、供应链创新与应用，加快建设10大价值创新园区，培育发展6个千亿级产业集聚区。培育一批＂两高四新＂企业，发展壮大大数据、云计算、移动互联网、量子信息、石墨烯、3D打印、共享经济、跨境电商、新零售等新技术新产业新业态新模式，加快形成新的增长点。实施工业企业技术改造三年行动计划，扶植一批大中型企业做优做强和中小微企业加快成长。启动老旧工业园区升级改造三年行动计划，淘汰和退出低效产能，力争低效园区单位面积产出实现倍增。

推动现代服务业提质增效。完善促进现代服务业发展政策，大力发展信息、金融、商务、中介、节能环保等生产性服务业，发展教育培训、健康养老、法律服务等生活性服务业，促进高端和高附加值服务业集聚。推动设计产业发展，建设国际设计之都、时尚之都。推广＂政府＋商会＋企业＂共治共管商圈发展模式，推动实体零售创新发展，引导专业市场转型升级，发展电子商务，举办国际购物节、国际美食节等主题展贸展销活动。建立完善现代物流服务体系，大力发展绿色供应链、智慧物流、冷链物流。加快建设世界级旅游品牌，打造世界旅游名城和国际旅游目的地集散地。

激发各类市场主体活力。对所有企业一视同仁、平等对待。出台促进国资国企改革创新发展实施意见，建立以管资本为主的国资监管模式，改组组建国有资本投资公司，加快战略性重组整合，推动国有资本做强做优做大。开展上市企业＂二次混改＂和职业经理人试点。支持民营企业发展，落实促进民营经济发展20条，强化产权保护，放宽民间资本市场准入，畅通民间资本进入渠道。激发和保护企业家精神，构建＂亲＂＂清＂新型政商关系，政府部门要用真心、谋真招、出真力，努力为企业排忧解难。

强化金融服务实体经济功能。常态化办好重大项目融资对接会，引导金融机构加大对实体经济的支持力度。提高直接融资比重，新增10家上市公司和100家新三板挂牌企业。发展普惠金融，加大对小微企业、创新创业、＂三农＂等薄弱领域的金融支持，为市民提供智能化、便利化、个性化金融服务。重点抓好国际金融城、金融创新服务区、南沙现代金融服务区、绿色金融改革试验区、白鹅潭产业金融服务创新区等功能区建设。强化金融风险防控，建设金融风险监测防控中心，严厉打击非法集资，守住不发生系统性金融风险的底线。

优化供给结构和质量。坚持积极的财政政策，加大对重点领域、重点项目的支持力度。发挥投资对优化供给结构的关键性作用，继续实施攻城拔寨行动，大力推进398个重点项目建设，带动全社会固定资产投资超过6600亿元。出台招商引资优惠政策，举办国际投资年会等高端招商活动，引进一批世界500强、大型跨国公司和行业领军企业项目。着力在＂破＂＂立＂＂降＂上下功夫，推进150多户国有＂僵尸企业＂实质出清，落实降成本政策措施，确保全年为企业减负700亿元。全面加强质量、品牌、标准强市工作，大力提升产品、工程、服务、环境质量，创建2个全国知名品牌示范区。建设先进标准化体系，推进企业质量技术创新。做好全国第四次经济普查和＂十三五＂规划中期评估。研究建立推动高质量发展的指标体系、统计体系，以及IAB、NEM等新兴产业运行统计监测体系。

（二）建设国家科技产业创新中心。把创新作为引领发展的第一动力，扎实推进国际科技创新枢纽建设，着力在企业创新和产学研深度融合上下功夫，打造科技之城、创新之城、机遇之城。

加快广深科技创新走廊建设。发挥广州的引领带动作用，对接国家自主创新示范区建设，重点研究区域产业共建、科技资源开放共享、人才跨区域流动等政策措施，明确广州段走廊协同发展的合作机制和产业功能布局。以＂四核和十三个创新节点＂为重点，加快建设科学城、生物岛、大学城－国际创新城、南沙庆盛科技创新创业基地等重大创新平台。深化中新（广州）知识城建设，打造知识密集型产业集聚区、创新要素集聚区和国际创新合作平台。推进琶洲互联网创新集聚区项目建设，促进互联网产业链价值链创新链拓展延伸。

培育壮大科技创新型企业。分层分类服务科技创新企业，建立覆盖种子期、初创期、成长期、成熟期的科技企业培育体系。开展高新技术企业树标提质行动，推动一批规模以上工业企业升级为高新技术企业，推动一批高新技术企业壮大为规模以上企业。做强做优做大科技创新企业，培育创新龙头企业和＂独角兽＂企业，重点扶持200家高精尖中小企业，新增小巨人企业1500家、科技创新企业1万家。

提升高精尖领域创新能力。突出关键共性技术、前沿引领技术、现代工程技术、颠覆性技术创新，形成一批领跑并跑的原始创新成果，抢占全国乃至全球的创新制高点。谋划一批重大科技基础设施和国家重点实验室建设，建设好再生医学与健康等省实验室、国家高分子材料产业创新中心。加大广州超算中心推广应用力度，建设南海可燃气体综合开发利用基地，争取更多国家重大科技专项布局广州。推动企业建设研发机构，规模以上工业企业研发机构比例达到45％。促进科技成果转化，推动高校和科研机构科技成果使用权、处置权、收益权改革。建设华南技术转移中心。设立科技成果产业化引导基金，扩大科技型中小企业信贷风险资金池规模，发展天使投资、风险投资、创业投资。

优化创新创业环境。优化完善＂1＋9＂政策，发挥财政资金杠杆作用，引导提高全社会研发投入。加快形成科技企业孵化育成体系，推动众创空间专业化发展。办好创交会、海交会、小蛮腰科技大会、官洲国际生物论坛、中以机器人大会、《财富》国际科技头脑风暴大会等高端创新会议。创建国家知识产权强市，开展知识产权运用和保护综合改革试验，推进国家版权贸易基地、商标品牌创新创业基地和中国（广东）知识产权保护中心建设。倡导创新文化，促进大众创业、万众创新上水平。

建设创新创业人才高地。深入实施产业领军人才＂1＋4＂政策。制定鼓励海外人才来穗创业红棉计划、促进人才服务机构创新发展和高层次人才岭南英杰工程、高技能人才羊城工匠计划，吸引全球顶尖人才、技术在广州集聚，建立外籍归国高层次人才创办内资企业、职称评审、出入境和居留等绿色通道。优化人才服务，探索人才绿卡前置发放，做好住房、子女教育等方面的保障，提供创新创业的广阔舞台，用事业聚人，用发展聚人。

（三）加快构建开放型经济新体制。弘扬敢为人先的改革精神，深度参与＂一带一路＂建设，借鉴吸收国内外先进经验，放大《财富》全球论坛效应，以开放促改革，推动形成全面开放新格局。

发挥粤港澳大湾区核心增长极作用。落实粤港澳大湾区发展规划，争取一批重大项目纳入国家粤港澳大湾区规划年度计划。加快推进一批跨区域交通基础设施建设。依托南沙粤港澳全面合作示范区，建设粤港产业深度合作园等重大产业合作平台。推动穗港澳合作，深化医疗、高等教育、科技创新、法律服务、会计服务、知识产权保护等领域政策对接，吸引港澳现代服务业企业落户。推进穗港澳服务贸易自由化，促进穗港澳人流、物流、信息流、资金流的互联互通。

打造南沙高水平对外开放门户枢纽。推动落实南沙自贸试验区更大改革自主权，加大制度创新力度，推进跨境人民币资金业务、商事登记和证照分离等方面先行先试。积极争取自由贸易港政策。加快国际中转和离岸贸易发展，吸引外贸机构在南沙设立商贸代表处，建设商品集散分拨中心。发展航运金融、贸易金融、供应链金融和风险投资，争取开展本外币账户管理创新试点，推动国际金融岛建设。

建设国际交往中心。出台建设国际交往中心三年行动计划。加强国际城市交往，新增一批友好城市和友好机构，深化与外国驻穗领馆等机构友好合作。打造国际交流合作平台，筹建广州国际交流合作中心，办好广交会、世界航线发展大会、第四届广州奖等重大活动，建设20个国际旅游推广中心。开展海外展示活动，积极参与对外商务推介展览，推动文化对外交流。营造国际化营商环境，发挥侨务优势，引导海外侨胞到广州投资兴业。完善国际标识系统，优化多语种服务平台，提升公共服务国际化水平。

率先加大营商环境改革力度。充分发挥市场在资源配置中的决定性作用，更好地发挥政府作用。出台实施优化营商环境政策，创建营商环境建设示范城市。对接国际投资贸易规则，推行外商投资准入前国民待遇加负面清单管理模式，深入推进通关一体化改革，优化整合海关特殊监管区域，高标准建立全球质量溯源中心。继续深化放管服改革，完善政府部门权责清单，推进行政审批制度改革创新试点，进一步推动建设工程项目审批改革，开展提升办理建筑许可便利度专项行动，清理规范行政审批中介服务。深化＂多证合一＂，开展商事登记服务跨境通，完善＂双随机一公开＂监管。

推动外经贸转型升级。出台新一轮参与＂一带一路＂建设三年行动计划。加强与沿线国家产能合作，参与沙特吉赞经济城、马六甲皇京港临海工业园、非洲吉布提自贸区等海外园区建设。创新海铁空联运模式，推进铁路国际货运班列发展。建设中小企业先进制造业中外合作区，推动本地制造业企业对接境外创新资源。发展市场采购、融资租赁等外贸新业态，建设跨境电商综合试验区。加大先进技术和设备、关键零配件和资源性产品进口。推动广货品牌＂丝路行＂，建设广州龙头企业海外生产销售基地。

加强区域合作。坚持服务周边就是服务自己，加强泛珠区域、高铁沿线经济带合作，推进产业融合、资源共享、旅游联盟。深入推进广佛同城化、广清一体化、广佛肇清云韶经济圈建设，协同建设广清产业合作园、北江引水工程、地铁7号线西延线等重大项目。加强与东莞、中山的战略合作，重点做好规划、交通基础设施、航运、生态、公共服务等方面对接。做好毕节、黔南、疏附、波密、甘孜、巫山、齐齐哈尔和梅州、清远对口帮扶、对口支援、对口合作，建立长效脱贫机制，坚决打赢脱贫攻坚战。

（四）建设枢纽型网络城市。围绕发挥国家重要中心城市引领带动作用，优化提升城市功能品质，在推动建设全球城市上迈出更加坚实有力的步伐。

优化城市发展布局。认真做好新一轮城市总体规划的报批实施，落实多规合一，完善管理平台，加快审批改革，以规划转型推动城市建设和治理转型。开展全国土地利用总体规划编制试点工作。完善一批城市重大发展平台控规。强化土地节约集约利用，加大土地收储力度，精准供给住宅用地、工业用地和重大基础设施用地，加大批而未供和闲置土地处置力度。

提升国际航运航空战略枢纽能级。制定实施新一轮国际航运中心建设三年行动计划。推进广州港深水航道拓宽工程、南沙港区四期工程、南沙国际邮轮码头等6个港口工程项目建设。以广州港为龙头整合优化周边地区沿江沿海港口资源，打造世界级枢纽港区。推进白云机场三期扩建等11个国际航空枢纽工程建设，启用白云机场第二航站楼，加快推进白云机场第四、第五跑道和第三航站楼报批，做好第二机场规划、征地拆迁等前期准备工作。全面启动临空经济示范区起步区开发，建设机场综合保税区二期项目，推动航空总部、航空物流、航空维修、航空制造和航空金融等临空产业集聚发展。

完善大交通网络体系。编制新一轮交通发展战略规划，推进5个国铁项目、6个城际轨道项目、9个地铁项目、10个高快速公路项目和100个市政道路工程建设。加快白云机场、广州北站空铁联运体系建设。新开工如意坊隧道、芳村大道南快捷化改造等27个道路工程，争取新开工建设棠溪站、深茂铁路、穗莞深城际琶洲支线等9个轨道项目，实现4条（段）地铁开通运营、通车总里程力争突破500公里。创建国家＂公交都市＂，建设中心区与外围区快速公交系统，实施停车场条例，加强出租车、网约车、共享单车、共享汽车管理。

加强重点功能区建设。推进主城区优化升级，有序疏解非中心城区功能，建设总部、金融、科技创新服务集聚区。加快建设南沙自贸试验区，打造广州城市副中心。推进外围城区扩容提质，重点打造知识城、东部和北部综合门户枢纽、南站商务区、北部生态绿洲，三个开发区优先发展先进制造业和战略性新兴产业，推动形成多点支撑的发展格局。优化提升一江两岸三带，推动珠江新城、国际金融城、琶洲互联网创新集聚区黄金三角区融合发展，打造三个十公里精品珠江，继续推进珠江两岸贯通工程，加快珠江国际慢岛规划建设。完善城市功能配置，推进电网、天然气管网、海绵城市、地下综合管廊和停车场充电桩建设。

有序推进城市更新改造。坚持成片连片更新和微改造协同并进，市、区联动推进系统化、差异化、网络化的城市更新。制定实施老旧小区改造行动计划，完成200个老旧小区改造，加快＂三线＂整治，推进旧楼宇加装电梯，建设城市慢行系统，推动老城区废旧空间改造利用，重点促进越秀、荔湾等老城区控量提质。继续推进13条旧村全面改造、3条旧村综合整治，力争冼村、田心村、大坦沙改造有突破性进展。推动19个旧厂房转型升级，促进低效存量建设用地再开发。完成6平方公里＂三旧＂改造任务。推进传统中轴线提升建设，加大历史文化街区和历史建筑保护。

（五）实施乡村振兴战略。着眼解决城乡发展不平衡不充分的问题，坚持农业农村优先发展，制定实施乡村振兴战略三年行动计划，推动农村加快实现产业兴旺、生态宜居、乡风文明、治理有效、生活富裕。

大力发展都市现代农业。培育壮大专业合作社和农业龙头企业，新建一批特色农产品生产基地，创建一批畜禽和水产养殖示范场，构建优质农产品供应体系。发展乡村旅游、农村电子商务、休闲农业等新业态，促进农村一二三产业融合。实施一批重点农业科技项目，申报建设国家种业科技创新中心，办好广州种业学院。推动蔬菜、花卉和种业等参保。落实粮食安全责任制，增强粮食保供稳价能力。培育新型职业农民，增加农民工资性收入和财产性收入。发展壮大海洋经济，培育发展海洋新兴产业。

不断深化农村改革。推进农村集体产权制度改革，基本完成农村土地承包经营权确权登记颁证。完善承包地＂三权分置＂制度，建立农村土地信息系统数据库，推进数据库与农村集体＂三资＂交易管理平台结合，开展农村土地流转竞价交易，引导土地经营权有序流转。推进农业补贴政策改革，保障农民财产权益，壮大集体经济。

加强农村基础设施建设。制定新一轮农村公路三年行动计划，设立路长制，切实把公路建好管好护好运营好。推进农村人居环境整治，突出抓好村道、水利、光纤网络、生活污水处理、垃圾处理、图书室、卫生站、金融服务站等设施建设，抓紧推进北部片区自来水供应工程。加快特色小镇创建，实施产城融合工程。促进北部地区新农村建设，巩固市内扶贫开发成果。建设生态宜居乡村环境，创建一批美丽乡村，连片打造美丽乡村群。

（六）建设美丽广州。牢固树立和践行绿水青山就是金山银山的理念，以更大决心更大力气打赢污染防治攻坚战，推动绿色发展，让广州更有魅力、更加宜居宜业宜游。

巩固提升＂天更蓝＂成果。强力推动中心城区污染企业彻底关闭或搬迁。深化工业燃煤污染治理，推广清洁能源应用，全面完成自备锅炉超洁净排放改造。强化机动车船等移动源污染控制，全市基本实现公交电动化。推动船舶使用低硫油和强制使用岸电。推进挥发性有机物污染防治，综合整治挥发性有机物重点企业和场所。强化扬尘管控，强力推行绿色施工。确保PM2.5稳定达标。

推进水污染防治。抓紧实施治水三年行动计划，继续全面推行河长制，推开实施湖长制。强化水污染执法，深入开展＂四洗＂行动，清理各类污染源和＂散乱污＂场所。巩固河涌治理成效，确保35条已治理河涌长治久清。强化源头治理，＂一河一策＂推进16条广佛跨界河涌和152条黑臭河涌综合整治，推进水环境综合治理向边沟边渠延伸。推进97条城中村、100条行政村生活污水治理，开工建设6座污水处理厂，建成1500公里污水管网。继续推进3个饮用水工程，城市集中式饮用水源地水质达标率稳定在100％。

推行垃圾分类处理。落实生活垃圾分类管理条例。实施生活垃圾强制分类，加大执法力度，健全激励惩戒机制，再创建300个生活垃圾分类样板居住小区（社区）。完善生活垃圾分类收集、分类运输体系。推动建筑废弃物资源化利用。启动新一轮垃圾处理设施建设。建成投产5个资源热力电厂和兴丰应急填埋场一期，新增垃圾焚烧日处理能力1.1万吨，城镇生活垃圾无害化处理率力争达到100％。

全方位提升生态建设水平。严守生态保护红线、永久基本农田、城镇开发边界三条控制线，实施生态环境损害赔偿制度，推进建立市场化、多元化生态补偿机制。强化土壤污染管控和修复，加强环境噪声、重金属、化学品等污染防治。完善绿色生态网络体系，提升园林绿化品质和公园服务功能，推进广州花园、城市主干道和主要出入口景观建设，种植10万株主题花树，新建生态景观林带40公里、绿道100公里、湿地公园1个、森林公园2个，让花城品牌更加响亮。

建设更干净更整洁更平安更有序城市环境。开展内街巷、城中村和农村环境治理，创建30个市级容貌示范社区、11条市容环境卫生责任区示范街镇，坚决遏制新增违法建设，依法处理历史违法建设。加强地下管线综合管理，新增管道燃气用户35万户。新改扩建一批公共厕所，把＂厕所革命＂拓展到城乡全域。健全应急管理体系，推进救灾物资储备中心建设。完善安全生产治理体系，建设安全教育体验中心，抓好重点行业领域安全监管，落实安全生产主体责任，完善消防设施，坚决遏制重特大安全事故。完善现代社会治安防控体系，提升应急处突能力，有效维护国家安全，严密防范暴力恐怖活动，严厉打击黄赌毒黑拐骗等违法犯罪活动，努力把广州建设成为最安全稳定、最公平公正、法治环境最好的地区。

（七）提高保障和改善民生水平。永远把人民对美好生活的向往作为奋斗目标，把老百姓的安危冷暖时刻放在心上，以造福人民为最大政绩，坚持在发展中保障和改善民生，加强和创新社会治理，努力办好民生实事，不断提高人民群众的获得感、幸福感、安全感。

优先发展教育事业。扩大基础教育学位供给，新增学前教育学位3.5万个，新改扩建中小学163所，新增学位13万个，再建市级示范性高中10所，完成校园微改造项目37个。推进互联网＋教育管理信息化和智慧校园建设，推行集团化、学区化办学，推动中心城区优质教育资源向外围城区和农村地区覆盖。加快职业教育和高等教育内涵式发展，推进广州大学、广州医科大学高水平大学建设，推动广州科技教育城、华南理工大学广州国际校区建设，筹建广州交通大学、旅游（烹饪）职业技术学院、幼儿师范专科学校。完善服务高层次外籍人才子女教育体系，筹建2所外籍人员子女学校。推动教育家培养工程，深化百名校长名校跟岗访学计划，引进基础教育高层次人才。支持和规范民办教育，发展网络教育，办好老年教育。规范中小学招生录取工作，完善学生校内课后托管机制，把来穗人员随迁子女入读义务教育公办学校（含政府补贴的民办学校）起始年级的比例提高到60％，着力解决儿童早期教育服务、＂择校热＂等突出问题，努力让每个孩子都能享有公平而有质量的教育。

提高就业质量。坚持就业优先战略和积极就业政策，推动实现更高质量和更充分就业。鼓励创业促就业，建设大学生创业孵化基地和创业园区。实施全民技能培训计划。新增城镇就业20万人，帮扶6万名就业困难人员实现再就业。健全劳动关系矛盾调处机制，构建和谐劳动关系。

推动文化事业和文化产业发展。培育和践行社会主义核心价值观，挖掘好保护好利用好红色革命遗址，深化文明广州建设。推进特色新型智库建设，建设社科创新之城。实施文化、学术精品创作工程，打造有影响力的文化品牌。推进文物史迹修缮保护，推动南粤古驿道、＂最广州＂文化步径等线性历史文化遗产连接和活化利用，牵头做好海上丝绸之路申遗工作。举办文交会等系列国际性文化活动，加快文化馆、美术馆、海事博物馆、国家档案馆新馆等重大文化设施建设，南汉二陵博物馆建成开馆。实施文化创意产业扶持计划，推动文化龙头企业上市。推进文化与科技、金融、旅游、商贸融合，扎实推进国家级文化产业示范园区创建工作。办好一批重大体育赛事，开展群众性体育活动，创新体育场馆惠民模式，发展体育产业，建设体育名城。

建设健康广州。继续推动卫生强市建设，加快构建医疗卫生高地，加强重点临床专科、卫生信息化、智慧医疗体系和高水平医院建设。推进医疗、医保、医药联动改革，深化公立医院改革，完善医保政策和药品供应保障，动态调整医疗服务价格，构建现代医院管理制度。深入推进分级诊疗、紧密型医疗共同体建设，家庭医生签约覆盖率30％以上、重点人群60％以上。强力推进强基创优、提升妇幼健康服务能力和加强精神卫生体系建设三年行动计划，抓好全科医生培养。实施基层中医药服务能力提升工程，传承发展中医药事业。健全传染病监测和防治体系，做好登革热等突发和重大传染病疫情防控。稳妥实施＂全面两孩＂政策。创建国家食品安全示范城市，推进肉菜市场升级改造，加强农产品和食品药品安全监管，让市民群众吃得放心。

加强社会保障体系建设。加快建立多主体供应、多渠道保障、租购并举的住房制度，保持房地产市场调控政策连续性和稳定性，完善促进房地产市场平稳健康发展的长效机制。增加保障房供应，建立健全政策性住房建设运营管理机制，支持专业化住房租赁企业发展，基本建成保障房1.6万套，新开工棚户区改造住房7500套。加大全自持租赁房、轨道交通沿线和集体建设用地上的租赁住房供应，让市民群众住有所居。完善社会保险体系，稳步提高待遇水平，落实企业职工养老保险省级统筹，推进机关事业单位养老保险制度和按病种分值付费改革，继续推进异地就医结算。鼓励社会资本举办养老机构，优化社区居家养老服务。完善＂中心城区10－15分钟、外围城区20－25分钟＂的养老助餐配餐服务网络，推动医疗、护理、心理等服务融入配餐网络。兜住民生底线，试行商业保险补充长期护理保险，推行社会救助精准服务，推进受助人员安置中心建设，创建慈善之城，雪中送炭、纾难解困，让广州更加温暖、更有人情味。

打造共建共治共享的社会治理格局。加强镇政府服务能力建设。创建幸福社区，发展社区服务综合体，推动社区、村议事厅建设全覆盖。实施＂社工＋＂战略，发展社区社会组织，让居民在家门口享受精细化服务、品质化生活。全面推进婚姻登记全城通办服务。持续推进来穗人员融合行动，开展出租屋专项整治，完善积分制公共服务体系。加强普法长效机制建设和法治文化宣传，优化公共法律服务，完善社会矛盾纠纷多元化解机制。

加强城市民族工作，积极引导宗教与社会主义社会相适应。做好双拥优抚安置工作。推动军民融合深度发展。授予一批荣誉市民称号。扎实做好国防动员、人民防空、人口计生、海防、打私、打假、统计、应急、气象、地震、参事、文史、档案、保密、修志等工作。

（八）努力建设人民满意的服务型政府。我们要坚持为民情怀，坚持以人民为中心，真正为民用权、公正用权、依法用权、廉洁用权，切实做到对人民负责、受人民监督、让人民满意。

深入推进政府职能转变。统筹各类机构编制，深化放权强区改革，赋予区更多自主权。有序推进财政事权与支出责任划分改革，完善预算制度，深化全过程绩效管理，调整优化财政支出结构，确保对重点领域、重点项目的支持力度，压缩一般性开支。推行政务服务规范化和标准化，完善基层政务服务和12345政府服务热线等便民服务体系。加快推动数字政府建设，创新政府网站管理，建立完善系统互通、数据共享的信息管理服务平台，让企业和群众办事更加方便快捷。

坚持依法全面履职。加强法治政府建设，完善政府立法工作机制，加强重大行政决策、规范性文件、重大行政执法决定合法性审查。加大政务公开力度，构建决策科学、执行坚决、监督有力的权力运行机制，严格规范公正文明执法。依法接受市人大及其常委会的监督，自觉接受市政协的民主监督，认真办理人大代表议案建议和政协提案。坚持有事好商量，众人的事情由众人商量，听取各民主党派、工商联、无党派人士和人民团体的意见，自觉接受监察监督，主动接受社会和舆论监督。

营造风清气正的良好政治生态。严格执行党内政治生活若干准则、廉洁自律准则、问责条例等，落实中央八项规定实施细则精神，全面治理庸政懒政怠政和形式主义、官僚主义，持之以恒纠正＂四风＂。落实全面从严治党主体责任和监督责任，运用监督执纪＂四种形态＂和＂三个区分开来＂，让政府工作人员知敬畏、存戒惧、守底线。深化基层正风反腐专项治理，整治损害群众利益的不正之风和腐败问题。加强审计监督，开展领导干部自然资源资产离任审计，让干部习惯在受监督和约束的环境中工作生活。

攻坚克难狠抓落实。深入开展＂不忘初心、牢记使命＂专题教育，把习近平新时代中国特色社会主义思想刻在骨子里、融入血液中，增强＂八个本领＂，为新时代履行新使命提供坚实支撑。大力开展＂大学习、深调研、真落实＂活动，深入基层、深入一线，找准事关广州发展大事和老百姓生活小事的短板和症结，形成推进落实行动方案。对市委、市政府的决策部署，要明确时间表路线图，下足绣花功夫，以滚石上山、爬坡过坎的决心和意志，推动各项工作落到实处。每一位政府工作人员特别是领导干部，既要雷厉风行，又要久久为功，以钉钉子的精神做实做细做好各项工作，努力做人民满意的公务员。

各位代表，新时代要有新气象，更要有新作为。让我们紧密团结在以习近平同志为核心的党中央周围，在省委、省政府和市委的坚强领导下，不忘初心，牢记使命，锐意进取，奋勇争先，推动国家重要中心城市建设全面上水平，努力在决胜全面建成小康社会、加快建设社会主义现代化新征程上走在前列，为实现中华民族伟大复兴的中国梦作出新的更大贡献！
